# Research Progress of Coronavirus Based on Bibliometric Analysis

**DOI:** 10.3390/ijerph17113766

**Published:** 2020-05-26

**Authors:** Fei Zhai, Yuxuan Zhai, Chuang Cong, Tingyan Song, Rongwu Xiang, Tianyi Feng, Zhengxuan Liang, Ya Zeng, Jing Yang, Jie Yang, Jiankun Liang

**Affiliations:** 1 School of Medical Equipment, Shenyang Pharmaceutical University, Shenyang 110016, China; zhaifei1978@gmail.com (F.Z.); zyxllgg@163.com (Y.Z.); songty214@yeah.net (T.S.); xrwlove@163.com (R.X.); 2 Liaoning Medical Big Data and Artificial Intelligence Engineering Technology Research Center, Shenyang 110016, China; 3 Shenyang Health Service and Administrative Law Enforcement Center, Shenyang 110031, China; congchuang76@163.com; 4 School of Materials Science and Engineering, Tsinghua University, Beijing 100084, China; fty17@mails.tsinghua.edu.cn; 5 Pharmaceutical Materials Science and Engineering Laboratory, College of Pharmacy, University of Minnesota, Minneapolis, MN 55455, USA; liangzx0312@gmail.com; 6 School of Pharmacy, Shenyang Pharmaceutical University, Shenyang 110016, China; zengtracy_stu@163.com (Y.Z.); emljaneyang@163.com (J.Y.); Youngyuj@163.com (J.Y.)

**Keywords:** bibliometrics, coronavirus, SARS-CoV-2, COVID-19

## Abstract

Background: COVID-19 has become one of the most serious global epidemics in the 21st Century. This study aims to explore the distribution of research capabilities of countries, institutions, and researchers, and the hotspots and frontiers of coronavirus research in the past two decades. In it, references for funding support of urgent projects and international cooperation among research institutions are provided. Method: the Web of Science core collection database was used to retrieve the documents related to coronavirus published from 2003 to 2020. Citespace.5.6.R2, VOSviewer1.6.12, and Excel 2016 were used for bibliometric analysis. Results: 11,036 documents were retrieved, of which China and the United States have contributed the most coronavirus studies, Hong Kong University being the top contributor. Regarding journals, the *Journal of Virology* has contributed the most, while in terms of researchers, Yuen Kwok Yung has made the most contributions. The proportion of documents published by international cooperation has been rising for decades. Vaccines for SARS-CoV-2 are under development, and clinical trials of several drugs are ongoing. Conclusions: international cooperation is an important way to accelerate research progress and achieve success. Developing corresponding vaccines and drugs are the current hotspots and research directions.

## 1. Introduction

A coronavirus is a kind of single-strand RNA virus that has the largest genome. It exists widely in nature and only infects vertebrates. It was first isolated from chickens in 1937 [1]. The virus that led to the outbreak of pneumonia in Wuhan in December 2019 is the seventh human coronavirus (HCoV) that can infect humans, following HCoV-229E, HCoV-NL63, HCoV-OC43, HCoV-HKU1, severe acute respiratory syndrome coronavirus (SARS-CoV), and Middle East respiratory syndrome coronavirus (MERS-CoV). The International Committee on Taxonomy of Viruses (ICTV) named the virus SARS-CoV-2 (Severe Acute Respiratory Syndrome Coronavirus 2), and the World Health Organization (WHO) officially named the resultant pneumonia disease COVID-19 (Coronavirus Disease 2019) [2]. Although SARS-CoV-2 has a lower fatality rate than SARS-CoV, it is more infectious [3]. As soon as the SARS-CoV-2 strains were isolated, research work on tracking, detection, and vaccine and drug development of the coronavirus was immediately launched worldwide [4,5]. As of 10 April 2020, it has spread to 205 countries and regions around the world, with a total of 1,610,788 confirmed cases and a total of 95,878 deaths. The total confirmed cases in the United States, Italy, Spain, France, and Germany have all reached 120,000, and public health emergencies have been declared in all of these countries [6]. In this study, CiteSpace5.6.R2, VOSviewer1.6.12, and Excel2016 were used to analyze the coronavirus literature published since the outbreak of SARS in 2003. Through the analysis of the research capabilities of countries, institutions, and authors, and of the hotspots and frontiers of coronavirus research [7], references are provided for funding support of urgent projects and international cooperation among research institutions.

## 2. Materials and Methods

Literature retrieval was conducted via the Web of Science Core Collection: Citation Indexes on 10 April 2020. Owing to the fact that most of the coronavirus research began with the SARS outbreak in 2003, the timespan was set as 2003–2020 (Figure S1). The advanced search option was adopted, and the retrieval strategy was TS = (coronavirus or Middle-East-Respiratory-Syndrome or Severe-Acute-Respiratory-Syndrome or 2019-nCoV or COVID-19 or SARS-CoV-2). The language was restricted to English; the document type limited to article, letter, and review; and only “Science Citation Index Expanded (SCI-EXPANDED)—1900-present” was included.

CiteSpace5.6.R2 (Drexel University, Philadelphia, PA, USA), VOSviewer1.6.12 (Leiden University, Leiden, The Netherlands), and Excel2016 (Microsoft Corporation, Redmond, WA, USA) were used to carry out visual analysis of the publications. Excel was used to pre-process the data of the clustering table exported by CiteSpace and to draw the geographic density distribution maps. CiteSpace was then used to carry out the analysis of dual-map overlays of journals and keyword emergence, and VOSviewer used to analyze the co-citation network of authors, organizations, countries, and journals.

## 3. Results

### 3.1. Analysis of Trend of Annual Publications

**Figure 1:**
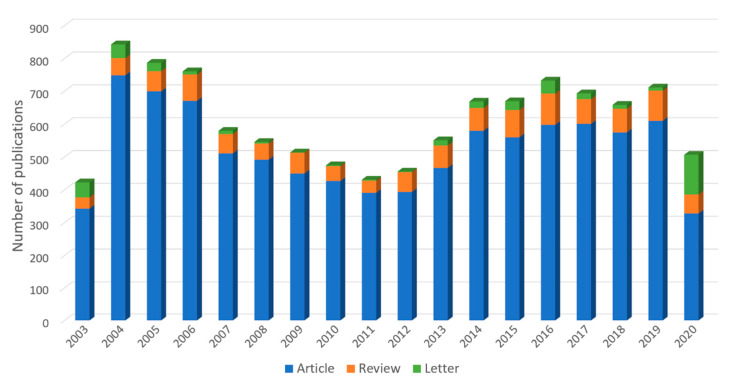
The trend of annual publications on the research of coronavirus during 2003–2020.

A total of 11,036 documents were retrieved, including 9459 articles (85.7%), 1173 reviews (10.6%), and 404 letters (3.7%); the growth trend of the annual publications is shown in Figure 1. The growth of publications showed a rising trend from 2003–2004 and from 2012–2016. Two prominent peaks occurred in 2004 and 2016, corresponding to the outbreaks of SARS-Cov in 2003 [8] and MERS-Cov in 2015, respectively [9,10]. This shows that SCI literature is usually largely published during the several years following an outbreak, and the proportion of letters is high in the same year.

### 3.2. Analysis of Category

The category analysis results with CiteSpace are shown in Figure S2. The size of a circle is in proportion to the amount of literature in the category, and the thickness of the lines is proportional to the relevance between different categories. The colors of the circles correspond to different years. The purple edge of a circle represents high betweenness centrality. According to the figure, *Virology* (2957, 30.3%), *Infectious Diseases* (1594, 16.4%), *Immunology* (1306, 13.4%), *Microbiology* (1182, 12.1%), *Veterinary Sciences* (1163, 11.9%), and *Biochemistry* & *Molecular Biology* (1004, 10.3%) were the top six research areas on the list of category analyses. Among all those disciplines, *Biochemistry* & *Molecular Biology* and *Immunology* show high betweenness centrality.

**Figure 2:**
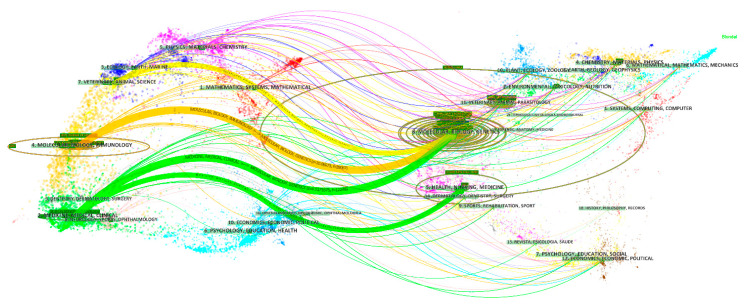
Dual map overlay of journals publishing research on coronavirus.

A dual map overlay of journals was used to analyze the dependence of the subject categories on coronavirus, and the results are shown in Figure 2. Citations made by these source articles are shown as spline waves, which are primarily rendered in yellow, green, and cyan. Each spline curve starts from a citing journal in the base map on the left and points to a cited journal in the base map on the right. Labels near the launching areas indicate the corresponding disciplines in which citing articles were published [11,12].

Figure 2 also shows that the journals containing coronavirus research are mainly distributed in three fields: virology (including *molecular*, *biology*, and *immunology*), infectious diseases (including *medicine*, *medical*, and *clinical*), and veterinary medicine. Many other disciplines are also involved, e.g., *chemistry*, *ecology*, *dentistry*, *dermatology*, *surgery*, and *ophthalmology*. There are four main citing paths from top to bottom, two yellow paths (starting from *veterinary science* and *virology*), and two green paths (both starting from *infectious disease*). The top three paths (starting from *veterinary science*, *virology*, and *infectious disease*) are mainly citing the literature of *molecular biology*, *biology*, and *genetics*. The fourth path (starting from *infectious disease*) mainly cites the literature related to *health care*, *nursing*, and *medicine*. This shows that *molecular biology*, *biology*, and *genetics* are the basis of coronavirus research.

### 3.3. Analysis of Authors

**Figure 3:**
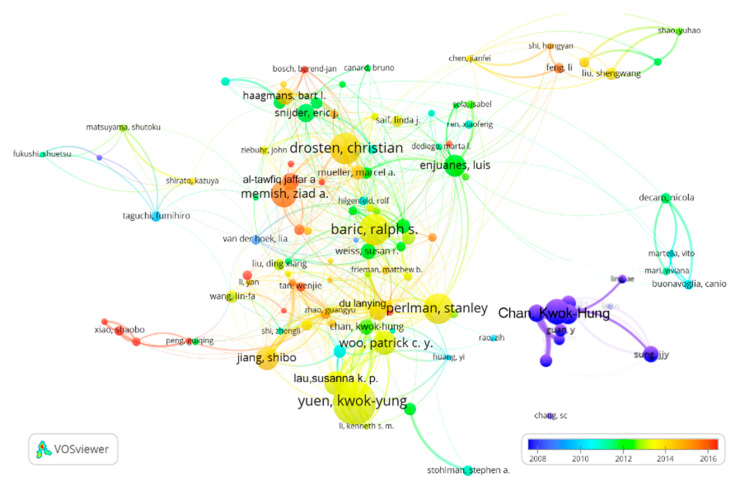
Co-authorship network of authors.

A collaboration network, shown as Figure 3, was analyzed for 114 authors who reached thresholds of 25 publications and 300 citations. The size of a circle is in proportion to the number of publications of the author, the color of a circle corresponds to the publication year, and the thickness of the lines is proportional to the cooperation frequency.

The comprehensive weight of each author was evaluated using Excel with the parameters derived from the collaboration network. The formula of the comprehensive weight is

comprehensive weight = weight of frequency + weight of citations + weight of h-index,

$$\mathrm{weight}\;\mathrm{of}\;\mathrm{frequency}=\frac{\mathrm{count}\;\mathrm{of}\;\mathrm{documents}-\mathrm{Min}\left(\mathrm{count}\;\mathrm{of}\;\mathrm{documents}\right)\;}{\mathrm{Max}\left(\mathrm{count}\;\mathrm{of}\;\mathrm{documents}\right)-\mathrm{Min}\left(\mathrm{count}\;\mathrm{of}\;\mathrm{documents}\right)}$$

$$\mathrm{weight}\;\mathrm{of}\;\mathrm{citations}=\frac{\mathrm{cumulative}\;\mathrm{citations}-\mathrm{Min}\left(\mathrm{cumulative}\;\mathrm{citations}\right)}{\mathrm{Max}\left(\mathrm{cumulative}\;\mathrm{citations}\right)-\mathrm{Min}\left(\mathrm{cumulative}\;\mathrm{citations}\right)}$$

$$\mathrm{weight}\;\mathrm{of}\;h-\mathrm{index}=\frac{h-\mathrm{index}-\mathrm{Min}\left(h-\mathrm{index}\right)}{\mathrm{Max}\left(h-\mathrm{index}\right)-\mathrm{Min}\left(h-\mathrm{index}\right)}$$

According to the comprehensive weight ranking, the top 10 most productive authors are listed in Table 1. Among them, five authors are from China, two from the USA, and one each from Germany, Saudi Arabia, and The Netherlands. The main research fields of the authors are microbiology, virology, immunology, infectious diseases, as well as biochemistry and molecular biology, among others. 

### 3.4. Analysis of Organizations

**Figure 4:**
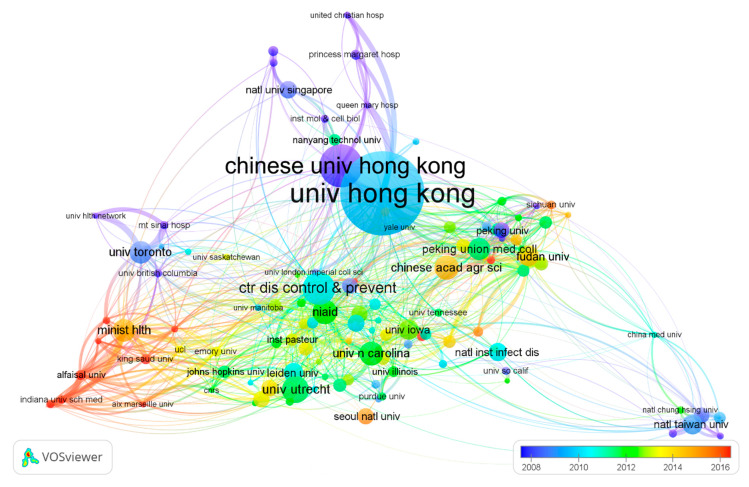
Co-authorship network of organizations. (Note: the size of a circle is in proportion to the number of documents of the organization, the color of a circle corresponds to the year, and the thickness of the lines is proportional to the cooperation frequency).

Figure 4 shows the collaboration network of 116 organizations that had over 40 publications and more than 400 citations. The top three most productive organizations were the University of Hong Kong (595), Chinese University of Hong Kong (311), and the National Institute of Allergy and Infectious Diseases (277). According to the comprehensive weights, the top 20 organizations in coronavirus research are listed in Table 2. Among them, seven organizations are from the USA; four from China; three from The Netherlands; and one each from Canada, the United Kingdom, Germany, Singapore, Taiwan, and Saudi Arabia. The total number of documents published by the top 20 organizations was 4006 (36.3%). The University of Hong Kong had the highest h-index (88), followed by the National Institute of Allergy and Infectious Diseases (66) and Chinese University of Hong Kong (55). The top three organizations ranked by collaboration are the University of Hong Kong, Centers for Disease Control and Prevention (CDC), USA, and the National Institute of Allergy and Infectious Diseases (NIAID). These all have cooperative relationships with almost all of the influential scientific institutions in the field of coronavirus research around the world (Figure S3).

### 3.5. Analysis of Countries/Territories

**Figure 5:**
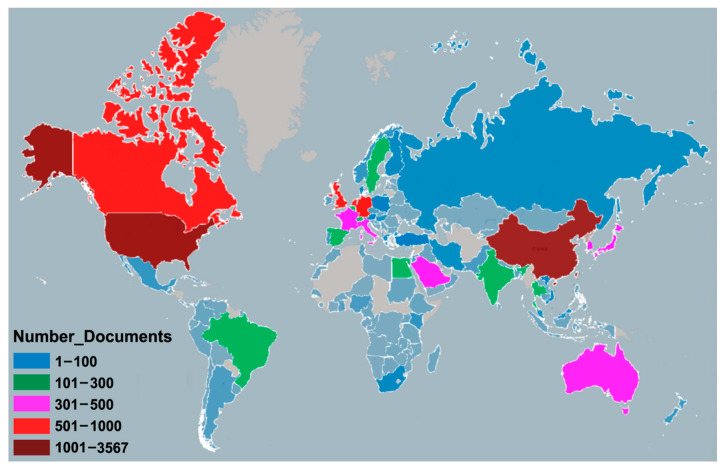
Geographical distribution of coronavirus publications. (Note: regions with no colors in the map have no available data).

Figure 5 shows the geographical distribution of coronavirus publications worldwide. The retrieved documents were contributed by researchers from 129 countries/territories.

**Figure 6:**
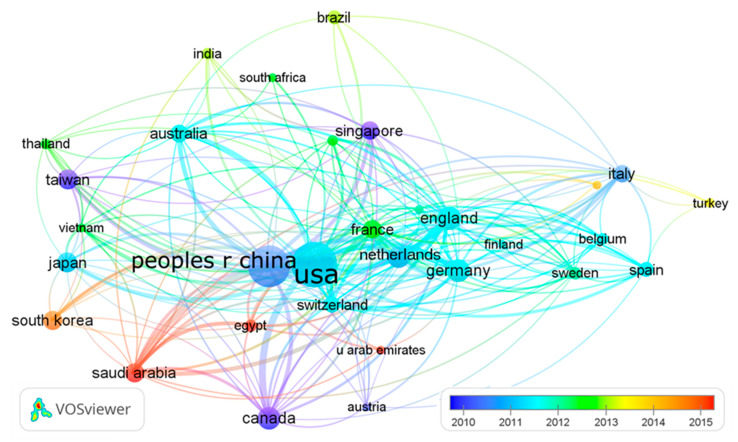
Collaboration network of countries.

Table 3 shows the 22 countries/territories with a minimum contribution of 100 publications ranked by comprehensive weight. Among them, nine countries/territories are from Europe, eight from Asia; two from North America; and one each from South America, Africa, and Australia. They contributed 10,561 (95.7%) publications in total. The USA ranked first in productivity with a total of 3606 publications (32.7%), followed by China (*n* = 3139; 28.4%); both contributed much more than third-place Germany (*n* = 669; 6.1%). For two decades, countries around the world have carried out extensive international cooperation on coronavirus research. The USA (95), Germany (81), England (81), France (73), and China (70) were the countries with the most partnerships in the world. Figure 6 shows inter-country collaboration among the countries, with the thickness of the lines representing the frequency of collaboration and the node color the publishing year. With the outbreak of SARS in 2003, China, Canada, Taiwan, Hong Kong, and Singapore took the lead in the coronavirus study; South Korea, Saudi Arabia, Egypt, and other countries began to carry out extensive research on coronavirus after the impact of the MERS outbreak in 2012 [13].

Funds provide important financial support for scientific research. Moreover, 5559 funding agencies from 129 countries provided funding support for the retrieved documents. Table 4 lists the top 10 funding agencies with the highest output. Among them, four funding agencies are from the USA; three from China; and one each from the European Union, Japan, and Germany. These countries contributed 5849 (53.0%) documents. The top two most productive funding agencies were the U.S. Department of Health and Human Services (1737) and U.S. National Institutes of Health (1682).

### 3.6. Analysis of Journals

**Figure 7:**
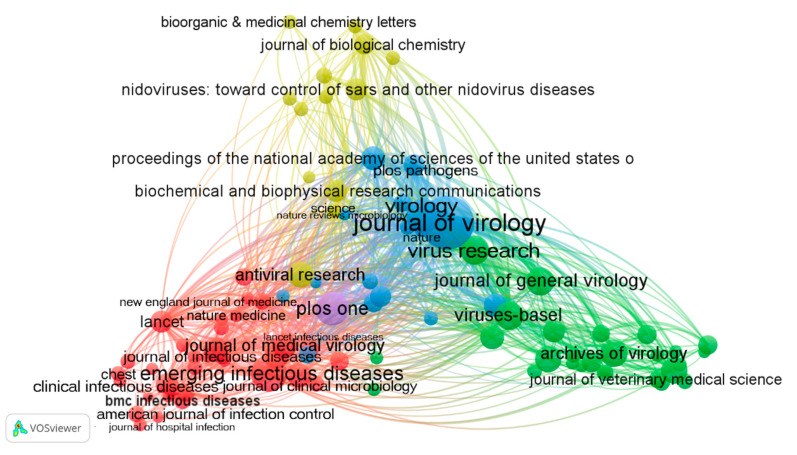
Documents and citation network of journals.

The retrieved documents were published in 1609 different journals, 93 having a minimum contribution of 20 documents and more than 200 citations (Figure 7). In the figure, the size of a circle is proportional to the number of documents published in the journal, the colors of circles represent different subject clusters, and a line represents a reference relationship.

According to the subject categories of the cited literature, these journals can be divided into four clusters from the macroscopic view. Figure 7 presents a triangle shape, with three discipline clusters at the corners and one cluster in the center. The journals in the yellow area on the top, represented by the *Journal of Biological Chemistry*, relate to biochemistry, pharmaceutical chemistry, molecular biology, bioorganic, and other basic disciplines, i.e., the cited literature mainly from the top and middle areas of the triangle. The journals in the red area in the lower left-hand corner represented by *Emerging Infectious Diseases* are related to the field of infectious diseases, and the citations mainly come from the lower left-hand corner, center, and lower right-hand corner. The journals in the green area in the lower right-hand corner, represented by *the Archives of Virology*, relate to the field of virology, with references mainly from the lower right-hand corner, center, and lower left-hand corner. The journals in the center blue area are the comprehensive journals represented by the *Journal of Virology*, of which the citations come from all four parts of the triangle.

Table S1 shows the top 20 journals in the field of coronavirus research ranked by comprehensive weight. They contributed 3452 (31.3%) publications in total. The *Journal of Virology* ranked first in productivity with a total of 826 (7.5%), followed by *Emerging Infectious Diseases* (306, 2.8%) and *Virology* (269, 2.4%). The *New England Journal of Medicine* and *Lancet* had the highest impact factors (IFs), 70.67 and 59.102, respectively, much higher than the IF value of the third place *Proceedings of the National Academy of Sciences of the United States of America* (9.58). In general, the higher the impact factor, the larger the citation per article.

### 3.7. Analysis of Keywords

**Figure 8:**
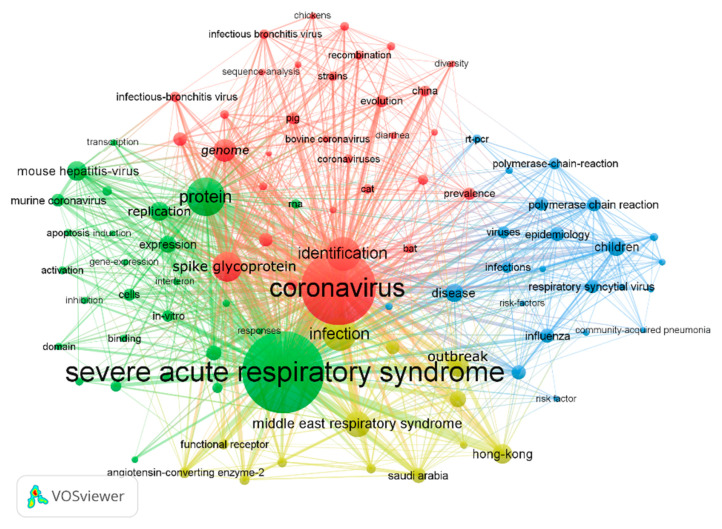
Co-occurrence network of keywords.

Figure 8 shows the co-occurrence network of keywords included in the retrieved documents. The node size represents the frequency of keyword occurrence in proportion, the thickness of a line the frequency of the two keywords co-occurrence in the same document, and the node colors the different clusters. Macroscopically, the keywords are divided into four clusters: top red region, left-hand green region, right-hand blue region, and lower yellow region. The top 10 keywords with the highest frequency are *Severe Acute Respiratory Syndrome* (4451), *Coronavirus* (4049), *Infection* (1657), *Protein* (1628), *Identification* (1248), *Spike Glycoprotein* (1020), *Middle East Respiratory Syndrome* (933), *Genome* (760), *Outbreak* (717), and *Replication* (563).

The literature of the red clusters mainly comes from the journals in *veterinary science* and *animal science*, including the natural host of coronaviruses, tracing of viruses, design of animal models, and so on. There is one kind of coronavirus, *Porcine Epidemic Diarrhea Virus* (PEDV), which has not been proved to infect humans. In the past two decades, three outbreaks caused by coronavirus have occurred among humans: two were naturally hosted by bats, and the other was intermediate hosted by dromedary camels [14]. Besides bats and camels, cattle, pigs, dogs, cats, chickens, rats, and other wild animals were also reported to be natural hosts [15,16].

The literature of the green clusters mainly comes from the journals in the fields of *microbiology*, *virology*, *immunology*, *molecular biology*, and *genetics*, including *protein structure*, *invasion mechanism*, *variation of virus*, and *vaccine development*. Researchers found that the key enzyme of coronavirus protein processing is the main protease of the virus, and the RNA virus has evolved unprecedented structural variation [17]. It was reported that coronavirus has two envelope proteins, S protein and M protein. S protein is the main antigen of receptor binding and cell fusion, while M protein participates in the bud and formation of the envelope and plays a key role in the assembly of the virus [18,19].

The literature of the blue clusters mainly comes from the fields of *clinical medicine*, *ecsomatics*, and *infectious diseases*, including the diagnosis of coronavirus, clinical symptoms, treatment, and epidemic spread. It was proved that PLx-RVP (PLx Multi-Code Respiratory Virus Panel) is a high-accuracy respiratory virus detection system based on experiments. Compared with traditional virus detection methods, it can significantly improve the detection accuracy of respiratory viruses [20]. Clinical data analysis showed that SARS coronavirus spread only by close contact, and the main symptoms were *fever* (100%), *dry cough* (100%), *dyspnea* (80%), and *lung lesions* (100%). These symptoms were accompanied with *lactate dehydrogenase increasing* (80%), *lymphocytopenia* (89%), *aspartate transaminase increasing* (78%) and *creatinine kinase increasing* (56%). The therapeutic drugs included *Lopinavir/Ritonavir*, *Chloroquine*, and *Glycyrrhizin*, among others [21,22].

The literature of the yellow clusters is relatively scattered and is mainly related to the outbreak of MERS in Saudi Arabia and South Korea. It was confirmed that MERS-CoV came from infected camels by whole-genome alignment (WGA) [23]. According to clinical research, males, the elderly, and diabetics were susceptible to MERS-CoV [24].

Figure S4 shows the top 20 keywords with the strongest burst strength. The keyword bursts started in 2003, and were *outbreak*, *severe acute respiratory syndrome*, *transmission dynamics*, *genome*, and *identification*. This shows that in the early days of the SARS outbreak, the genome of SARS-CoV was determined for identification, and spreading dynamics analysis of SARS-CoV was carried out. With the deepening of research, the keywords *coronavirus main proteinase* and *angiotensin converting enzyme 2* that were related to virus structure and intrusion mechanism appeared. The keywords of MERS began to burst when the MERS epidemic broke out in Saudi Arabia and South Korea.

### 3.8. Latest SARS-CoV-2 Research

**Figure 9:**
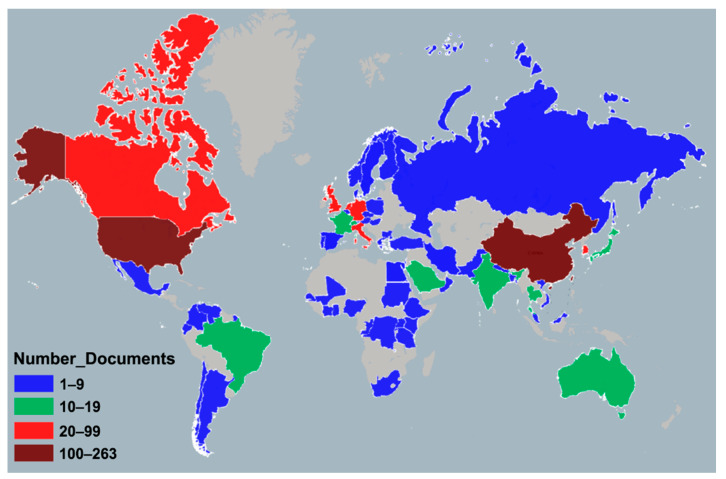
Geographical distribution of SARS-CoV-2 publications. (Note: regions with no colors in the map have no available data).

**Figure 10:**
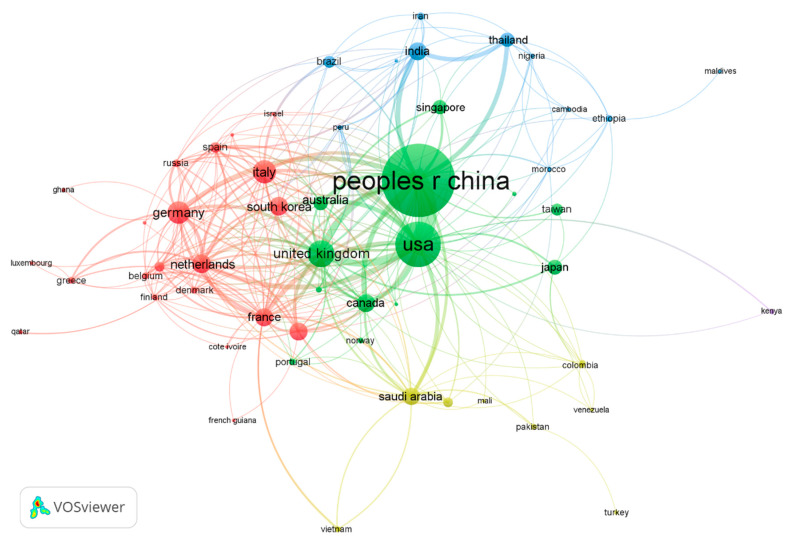
Co-authorship network of countries.

By 10 April 2020, 507 documents related to SARS-CoV-2 contributed by 56 countries/territories were published in the core collection of the Web of Science (WOS) (Figure 9). As shown in Figure 10, seven countries had more than 20 publications, including China (263), the USA (102), United Kingdom (40), Italy (31), Germany (24), South Korea (21), and Canada (20), which together contributed 501 (98.8%) publications.

The evolutionary history and cross-species transmission of SARS-CoV-2 were analyzed by whole genome sequence. The results showed that the original host of SARS-CoV-2 may be bats [4,25,26,27]. At present, in the absence of drugs specifically targeting SARS-CoV-2, social isolation measures (reducing aggregation, maintaining distance, wearing of masks, washing hands frequently, etc.) comprise effective preventive measures [28,29,30], and rapid and accurate detection methods were developed [31,32]. A variety of drugs, including Remdesivir, Favipiravir, chloroquine, hydroxychloroquine, Lopinavir/Ritonavir, are being tested in various countries [33,34,35,36]. Vaccines for SARS-CoV-2 are also being developed and have achieved varied results [37,38].

## 4. Discussion

Next to the outbreak of SARS, coronaviruses have caused wide concern and a significant amount of related research has been carried out. Researchers from 129 countries/territories have engaged in research work on coronaviruses, implying that coronaviruses have become a worldwide public health concern. The USA was the most productive country, which is not surprising since it has the largest number of scientific research institutions with significant research capacity, the largest number of P3 (Protection level 3) and P4 (Protection level 4) biosafety laboratories, the largest investment in scientific research funds, and the most extensive international scientific research cooperation in the world. Following the United States, China has also played a leading role in the field of coronavirus research, especially for SARS-CoV-2. This is related to the initial outbreak and isolation of the virus in China, which has a large number of scientific research institutions and a large amount of research funds.

This study shows that the proportion of documents published through international cooperation has been increasing for decades (Table S2). For the top 20 most productive countries, 30.6% of their publications had international cooperation before 2000, 46.7% in 2002–2010, and 58.2% in the most recent decade. Among these countries, 65% had a cooperation rate exceeding 50%, of which Egypt (94.5%) and The Netherlands (85.8%) had the largest proportion. This shows that international cooperation has become more important in coronavirus research.

The six documents cited over 1000 times were from the *New England Journal of Medicine* (70.67), *Lancet* (59.102), and *Science* (41.037), all of which are journals with higher IFs. Literature based on bibliometric analysis shows that, in general, IF is proportional to citation frequency [39].

Since the outbreak of COVID-19, more than 200 countries and regions have been affected in just three months. Research institutions around the world have carried out numerous coronavirus studies with respect to the tracing, structure, invasion mechanism, spreading, detection, prevention, and treatment of the virus. Although some progress has been achieved, more studies are needed to provide a basis for vaccine development and drug screening. There is no ultimate weapon to fight against a coronavirus except for a vaccine [40,41].

There are several limitations in our research. First, to obtain high-quality literature, the WOS Core Collection: Citation Indexes Database was used as the data source, the language limited to English, and document types limited to article, letter, and review, which leads a significant amount of coronavirus literature being excluded. Second, SARS-CoV-2 is a 3-month-old novel coronavirus, and the literature about it is emerging explosively. Therefore, the latest documents published after 10 April 2020, cannot be used and this may affect the results. Third, this study regards the contribution of multiple authors of a document as the same, which also leads to biased results in terms of the rank of countries, institutions, and authors.

## 5. Conclusions

Based on the Web of Science Core Collection, the literature on coronavirus research from 2003 to 2020 was analyzed using bibliometric methods. The number of publications on coronavirus research showed two clear peaks in the past two decades, one for SARS and the other for MERS. Owing to the global outbreak of COVID-19, a new peak of coronavirus research will appear in 2020. According to the results of the analysis, international cooperation is an important way to achieve success. The research institutions are mainly from China, the USA, and The Netherlands, and the universities are the most active institutions in scientific research. More than half of the studies were funded. The number of published papers and h-indexes showed that the USA, China, and Germany are the main contributors of high-quality papers. Bibliometric analysis may provide references for funding support of much-needed projects and international cooperation among research institutions [42]. At present, as a novel coronavirus, SARS-CoV-2 is spreading rapidly around the world. In the absence of drugs specifically targeting it, prevention is the most effective mitigation strategy. Seeking effective targets for SARS-CoV-2 and developing corresponding vaccines and drugs are the current hotspots and research directions.

## Figures and Tables

**Table 1:** Top 10 authors in coronavirus research.

CWR	Author	Organization	Frequency	Citations	h-Index
1	Yuen, Kwok-yung	University of Hong Kong (China)	214	17,507	70
2	Chan, Kwok-Hung	University of Hong Kong (China)	122	11,760	55
3	Drosten, Christian	University of Bonn (Germany)	142	11,069	48
4	Woo, Patrick C. Y.	University of Hong Kong (China)	119	7296	47
5	Lau, Susanna K. P.	University of Hong Kong (China)	113	6960	46
6	Baric, Ralph S.	University of North Carolina (USA)	131	5240	44
7	Memish, Ziad A.	Ministry of Health (Saudi Arabia)	100	5355	41
8	Perlman, Stanley	University of Iowa (USA)	122	3666	34
9	Snijder, Eric J.	Leiden University (The Netherlands)	83	5589	38
10	Jiang Shibo	Fudan University (China)	96	3217	34

CWR, comprehensive weight ranking; h-index, Hirsch index.

**Table 2:** Top 20 institutions in the field of coronavirus research.

CWR	Organization	Country/Territories	Frequency (%)	Citations	h-Index
1	The University of Hong Kong	China	595 (5.4%)	34,129	88
2	Chinese University of Hong Kong	China	311 (2.8%)	12,070	55
3	Centers for Disease Control and Prevention	USA	266 (2.4%)	13,443	55
4	Erasmus University Rotterdam	The Netherlands	163 (1.5%)	13,907	51
5	Utrecht University	The Netherlands	206 (1.9%)	8960	53
6	National Institute of Allergy & Infectious Diseases	USA	182 (1.6%)	9250	54
7	University of North Carolina	USA	209 (1.9%)	8083	52
8	University of Toronto	Canada	205 (1.9%)	8260	45
9	Harvard University	USA	143 (1.3%)	8267	47
10	Leiden University	The Netherlands	138 (1.3%)	7853	45
11	University of London	UK	147 (1.3%)	7367	44
12	University of Bonn	Germany	122 (1.1%)	7354	47
13	National University of Singapore	Singapore	161 (1.5%)	5111	35
14	National Taiwan University	Taiwan	180 (1.6%)	3839	35
15	Peking Union Medical College	China	175 (1.6%)	4752	32
16	Ministry of Public Health	Saudi Arabia	131 (1.2%)	4529	38
17	The University of Iowa	USA	141 (1.3%)	4165	37
18	China Centers for Disease Control and Prevention	China	156 (1.4%)	4806	33
19	Johns Hopkins University	USA	155 (1.4%)	4362	34
20	University of Pennsylvania	USA	133 (1.2%)	3994	36

CWR, comprehensive weight ranking; h-index, Hirsch index.

**Table 3:** List of countries/territories with a minimum contribution of 100 documents.

CWR	Country/Territory	Frequency (%)	TC	C/A	NPC	PDIC	h-Index
1	USA	3606 (32.7%)	127,036	35.2	95	44.6%	129
2	China	3139 (28.4%)	84,144	26.8	70	32.8%	112
3	Germany	669 (6.1%)	31,925	47.7	81	67.0%	89
4	The Netherlands	575 (5.2%)	32,535	56.6	65	75.7%	84
5	United Kingdom	658 (6%)	26,007	39.5	81	51.8%	78
6	Canada	617 (5.6%)	23,063	37.4	48	59.3%	74
7	France	407 (3.7%)	13,427	33.0	73	72.7%	60
8	Australia	347 (3.1%)	12,333	35.5	52	66.4%	57
9	Taiwan	504 (4.6%)	12,073	24.0	31	24.6%	48
10	Saudi Arabia	398 (3.6%)	12,869	32.3	57	72.1%	51
11	Singapore	394 (3.6%)	13,127	33.3	48	50.4%	50
12	Switzerland	253 (2.3%)	11,107	43.9	60	82.3%	57
13	Italy	373 (3.4%)	9596	25.7	58	43.4%	53
14	Japan	478 (4.3%)	9249	19.3	36	38.8%	43
15	South Korea	450 (4.1%)	6699	14.9	29	24.5%	41
16	Spain	241 (2.2%)	7930	32.9	49	58.6%	44
17	Sweden	133 (1.2%)	5699	42.8	56	72.3%	31
18	Belgium	127 (1.2%)	3538	27.9	46	55.2%	33
19	Brazil	197 (1.8%)	2134	10.8	38	34.4%	24
20	Thailand	119 (1.1%)	4032	33.9	42	62.8%	25
21	Egypt	139 (1.3%)	2546	18.3	43	94.2%	25
22	India	147 (1.3%)	1849	12.6	32	52.2%	22

CWR, comprehensive weight ranking; TC, total citations; C/A, citation per article; NPC, number of partner countries; PDIC, percentage of documents with international cooperation; h-index, Hirsch index.

**Table 4:** Top 10 funding agencies with highest output.

Rank	Funding Agency	Country	Documents	TC
1	U. S. Department of Health and Human Services	USA	1737	67,900
2	National Institutes of Health	USA	1682	66,579
3	National Institute of Allergy Infectious Diseases	USA	766	35,357
4	National Natural Science Foundation of China	China	685	9617
5	European Union	EU	175	5809
6	Ministry of Education Culture Sports Science and Technology of Japan	Japan	172	2493
7	German Research Foundation	Germany	130	5379
8	National Institute of General Medical Sciences	USA	124	6434
9	National Basic Research Program of China	China	120	2748
10	National Key Research and Development Program of China	China	114	888

TC, total citations; EU, European Union.

## Supplementary Materials

The following are available online at https://www.mdpi.com/1660-4601/17/11/3766/s1, Figure S1: The trend of annual publications on the research of coronavirus during 1969–2020, Figure S2: Co-occurring subject category network, Figure S3: Partial co-authorship network of organizations, Figure S4: Top 20 keywords with the strongest burst strength, Table S1: Top 20 journals in the field of coronavirus, Table S2: Proportion of documents published by international cooperation.
