# Supplementary material for: Research Progress of Coronavirus Based on Bibliometric Analysis
Source: Int J Environ Res Public Health. 2020 May 26;17(11):3766. doi: 10.3390/ijerph17113766 (PMC7312058; doi:10.3390/ijerph17113766)
Supplement: Supplementary file 1 [file ijerph-17-03766-s001.zip › Table S1. Top 20 journals in the field of coronavirus.docx]

**Table S1.** Top 20 journals in the field of coronavirus

| CWR | Journal | F(%) | C/A | IF | JCR^®^ Category(Quartile) | Country |
| --- | --- | --- | --- | --- | --- | --- |
| 1 | Journal of Virology | 826(7.5%) | 42.6 | 4.324 | virology(Q1) | USA |
| 2 | Emerging Infectious Diseases | 306(2.8%) | 40.5 | 7.185 | immunology(Q1), infectious diseases(Q1) | USA |
| 3 | Proc Natl Acad Sci Usa | 108(1.0%) | 108.5 | 9.58 | multidisciplinary sciences(Q1) | USA |
| 4 | Virology | 269(2.4%) | 25.9 | 2.657 | virology(Q3) | USA |
| 5 | Journal of General Virology | 171(1.5%) | 31 | 2.809 | biotechnology & applied microbiology(Q2), virology(Q2) | UK |
| 6 | Lancet | 66(0.6%) | 164 | 59.102 | medicine, general & internal(Q1) | UK |
| 7 | Plos One | 250(2.3%) | 18.7 | 2.776 | multidisciplinary sciences(Q2) | USA |
| 8 | Journal of Clinical Microbiology | 110(1.0%) | 45.2 | 4.959 | microbiology(Q1) | USA |
| 9 | Virus Research | 189(1.7%) | 23.9 | 2.736 | virology(Q3) | NL |
| 10 | Clinical Infectious Diseases | 90(0.8%) | 57.4 | 9.055 | immunology(Q1), infectious diseases(Q1), microbiology(Q1) | UK |
| 11 | Journal of Medical Virology | 132(1.2%) | 30.8 | 2.049 | virology(Q3) | USA |
| 12 | Journal of Infectious Diseases | 109(1.0%) | 37.7 | 5.045 | immunology(Q1), infectious diseases(Q1), microbiology(Q1) | UK |
| 13 | New England Journal of Medicine | 42(0.4%) | 257.6 | 70.67 | medicine, general & internal(Q1) | USA |
| 14 | Plos Pathogens | 83(0.8%) | 51.5 | 6.463 | microbiology(Q1), parasitology(Q1), virology(Q1) | USA |
| 15 | Journal of Biological Chemistry | 76(0.7%) | 53.8 | 3.95 | biochemistry & molecular biology(Q2) | USA |
| 16 | Biochemical And Biophysical Research Communications | 93(0.8%) | 36.5 | 2.705 | biochemistry & molecular biology(Q3), biophysics(Q2) | USA |
| 17 | Journal of Clinical Virology | 97(0.9%) | 29.5 | 3.02 | virology(Q2) | NL |
| 18 | Journal of Virological Methods | 144(1.3%) | 18.1 | 1.746 | biochemical research methods(Q4), biotechnology & applied microbiology(Q3), virology(Q4) | NL |
| 19 | Veterinary Microbiology | 139(1.3%) | 17.3 | 2.791 | microbiology(Q3), veterinary sciences(Q1) | NL |
| 20 | Archives of Virology | 152(1.4%) | 15.5 | 2.261 | virology(Q3) | Austria |

CWR comprehensive weight ranking, F(%) Frequency(%N=11036), C/A citation per article, IF impact factor, USA the United States of America, UK the United Kingdom, NL Netherlands, Proc Natl Acad Sci USA: Proceedings of the National Academy of Sciences of the United States of America
