# Supplementary figures and images for: Research Progress of Coronavirus Based on Bibliometric Analysis
Source: Int J Environ Res Public Health. 2020 May 26;17(11):3766. doi: 10.3390/ijerph17113766 (PMC7312058; doi:10.3390/ijerph17113766)

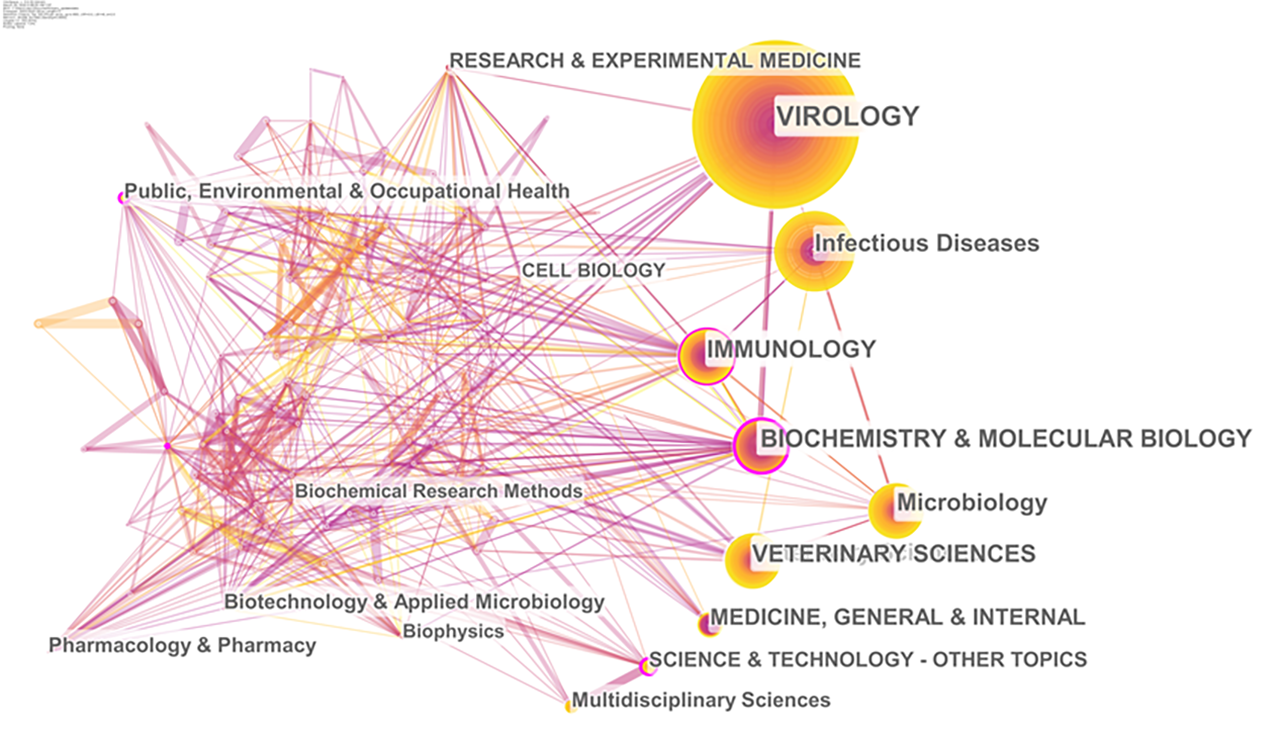

Supplement: Supplementary file 1 [file ijerph-17-03766-s001.zip › Figure S1. Co-occurring subject category network.png]

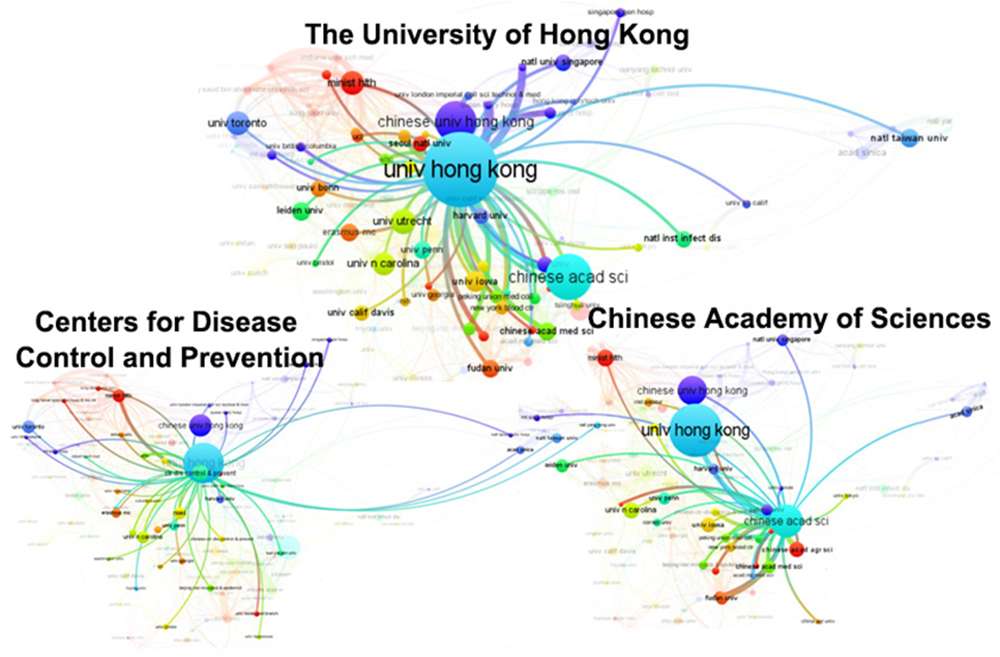

Supplement: Supplementary file 1 [file ijerph-17-03766-s001.zip › Figure S2. Partial co-authorship network of organizations.png]

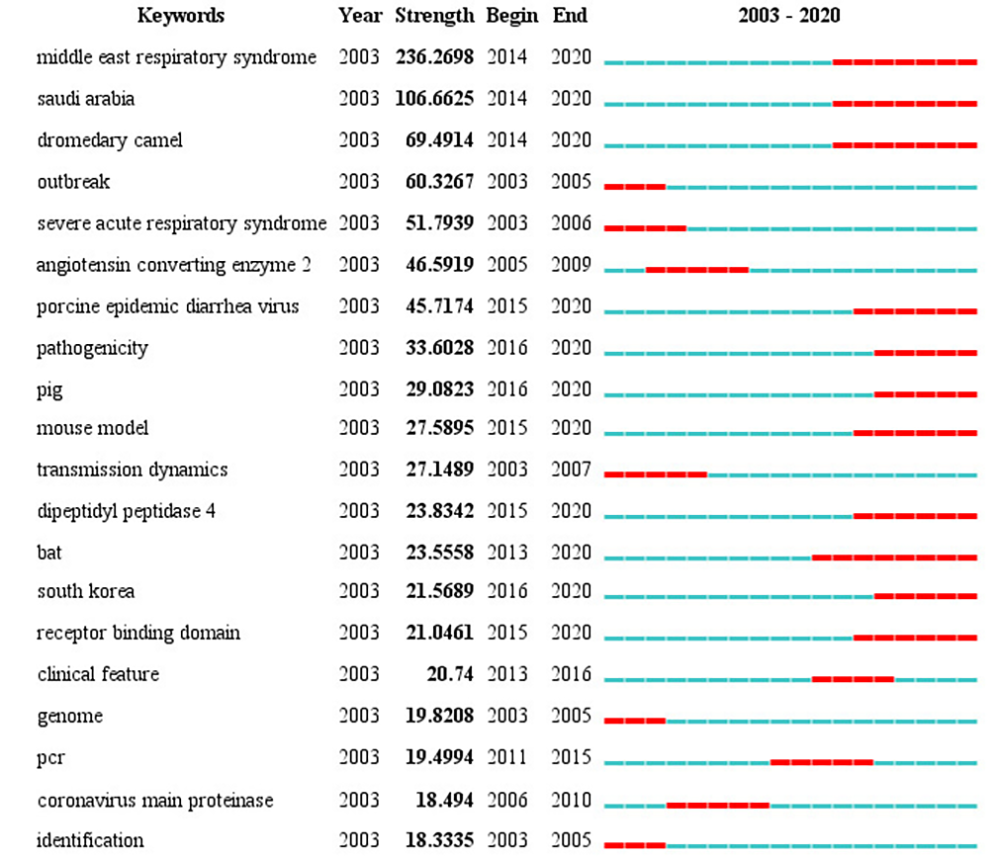

Supplement: Supplementary file 1 [file ijerph-17-03766-s001.zip › Figure S3. Top 20 keywords with the strongest burst strength.png]

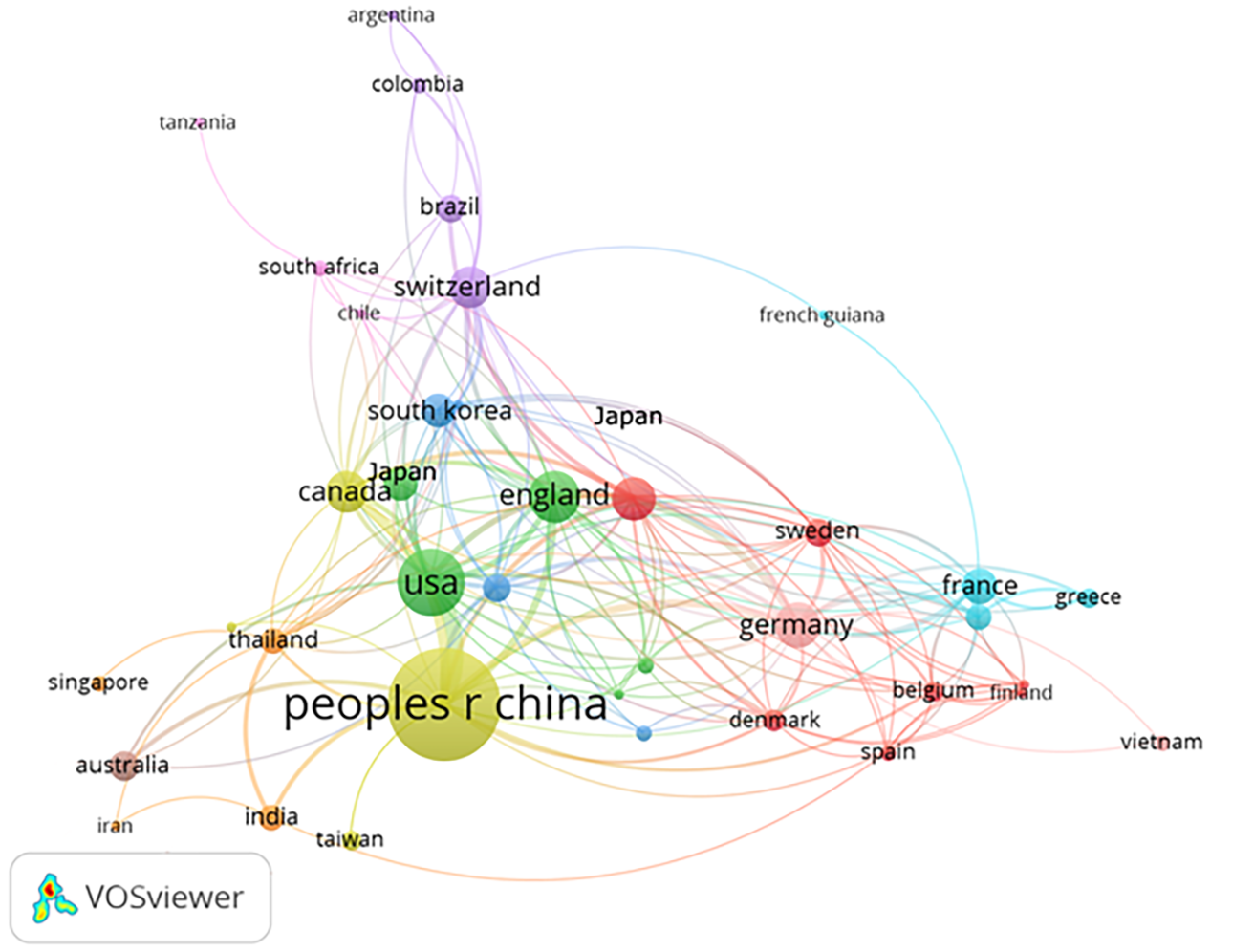

Supplement: Supplementary file 1 [file ijerph-17-03766-s001.zip › Figure S4. Collaboration network of SARS-CoV-2 research.png]
